# Skeletal open bite with amelogenesis imperfecta treated with compression osteogenesis: a case report

**DOI:** 10.1186/s13005-019-0187-7

**Published:** 2019-01-28

**Authors:** Hiroki Mori, Takashi Izawa, Hitoshi Mori, Keiichiro Watanabe, Takahiro Kanno, Eiji Tanaka

**Affiliations:** 1 Department of Orthodontics and Dentofacial Orthopedics, Institute of Biomedical Sciences, Tokushima University Hospital, Tokushima, Japan; 2 Department of Orthodontics and Dentofacial Orthopedics, Institute of Biomedical Sciences, Tokushima University Graduate School, 3-18-15 Kuramoto-cho, Tokushima, 770-8504 Japan; 3 Mori Dental & Orthodontic Office, Kagawa, Japan; 4 Department of Orthodontics and Dentofacial Orthopedics, Tokushima University Hospital, Tokushima, Japan; 5 Department of Oral and Maxillofacial Surgery, Shimane University Faculty of Medicine & Maxillofacial Trauma Center, Shimane University Hospital, Shimane, Japan; 6 Faculty of Dentistry, King Abdulaziz University, Jeddah, Saudi Arabia

**Keywords:** Open bite, Anchor plate, Compression osteogenesis, Corticotomy

## Abstract

**Background:**

We successfully treated a 37-year-old male who had skeletal open bite with severe amelogenesis imperfecta (AI) with orthodontics, compression osteogenesis, and prosthodontics.

**Case presentation:**

The patient was diagnosed with severe anterior open bite caused by severe AI. Corticotomy was performed on both buccal and palatal sides of the molar regions, and anchor plates were placed onto the bilateral zygomatic buttress and the center of the hard palate. After corticotomy, posterior maxillary segments were moved 3.5 mm superiorly to correct skeletal open bite with elastic chains. After 8-month, overbite had decreased by 2.0 mm. After further 5 months of prosthodontic preparation, orthodontic appliances were removed, and provisional crowns were set on all teeth. The anterior open bite was corrected, and ideal occlusion with a Class I molar relationship was achieved. The upper first molars were intruded 3.5 mm, resulting in 3.0^o^ counter-clockwise rotation of the mandible. The total active treatment period was 16 months. Acceptable occlusion with a good facial profile was well maintained throughout the 8-year retention period.

**Conclusions:**

Our results indicate long-term stability after interdisciplinary treatment combining orthodontics, oral surgery, and prosthodontics in a patient with severe anterior open bite and AI.

## Background

Amelogenesis imperfecta (AI) represents a group of developmental conditions, genomic in origin, that affect the structure and clinical appearance of enamel of all or nearly all teeth in a generally equivalent manner. AI is a serious issue that reduces oral health-related quality of life, with potentially significant aesthetic and physiological consequences. Treatment planning for patients with AI depends on many factors, including disorder severity, extent of destruction, age, and socioeconomic status. Treatment for AI may improve quality of life and self-esteem [1].

The most complicated dental issue encountered in adults is skeletal open bite [2]. A skeletal anchorage system was recently developed for treatment of severe open bite. Use of this system allows for correction of skeletal open bite without unfavorable side effects such as elongation of anterior teeth, resulting in significant relapse of open bite. However, some patients with severe open bite and skeletal discrepancy require surgical correction. The most effective treatment option for such patients may be surgical repositioning of the maxilla and/or mandible [3].

Compared with surgical osteotomy, corticotomy-facilitated orthodontic treatment does not seem to produce unwanted adverse effects on the periodontium, root resorption, and tooth vitality and decrease treatment time [4]. For example, compression osteogenesis has been used to treat jaw deformities [5, 6]. To treat adults with maxillary and/or mandibular protrusion, the anterior alveolar segment of the maxilla and/or mandible is moved posteriorly en bloc with a skeletal anchorage device, combined with corticotomy [5, 6]. This method has advantages similar to those mentioned above, such as a minimally invasive approach to surgery and marked shortening of the treatment period [5]. However, few case reports of skeletal open bite treated with compression osteogenesis have been published. In this study, we present an adult case of skeletal open bite with AI treated with compression osteogenesis.

## Case presentation

### Diagnosis and etiology

A 37-year-old male presented with masticatory disturbance and aesthetic complaints. His facial profile was straight, and the frontal view was almost symmetrical, with long lower facial height. When the patient smiled, upper incisors could not be seen below the upper lip. He exhibited no phenotypes and medical and family histories about osteogenesis imperfecta and bone diseases.

**Fig. 1:**
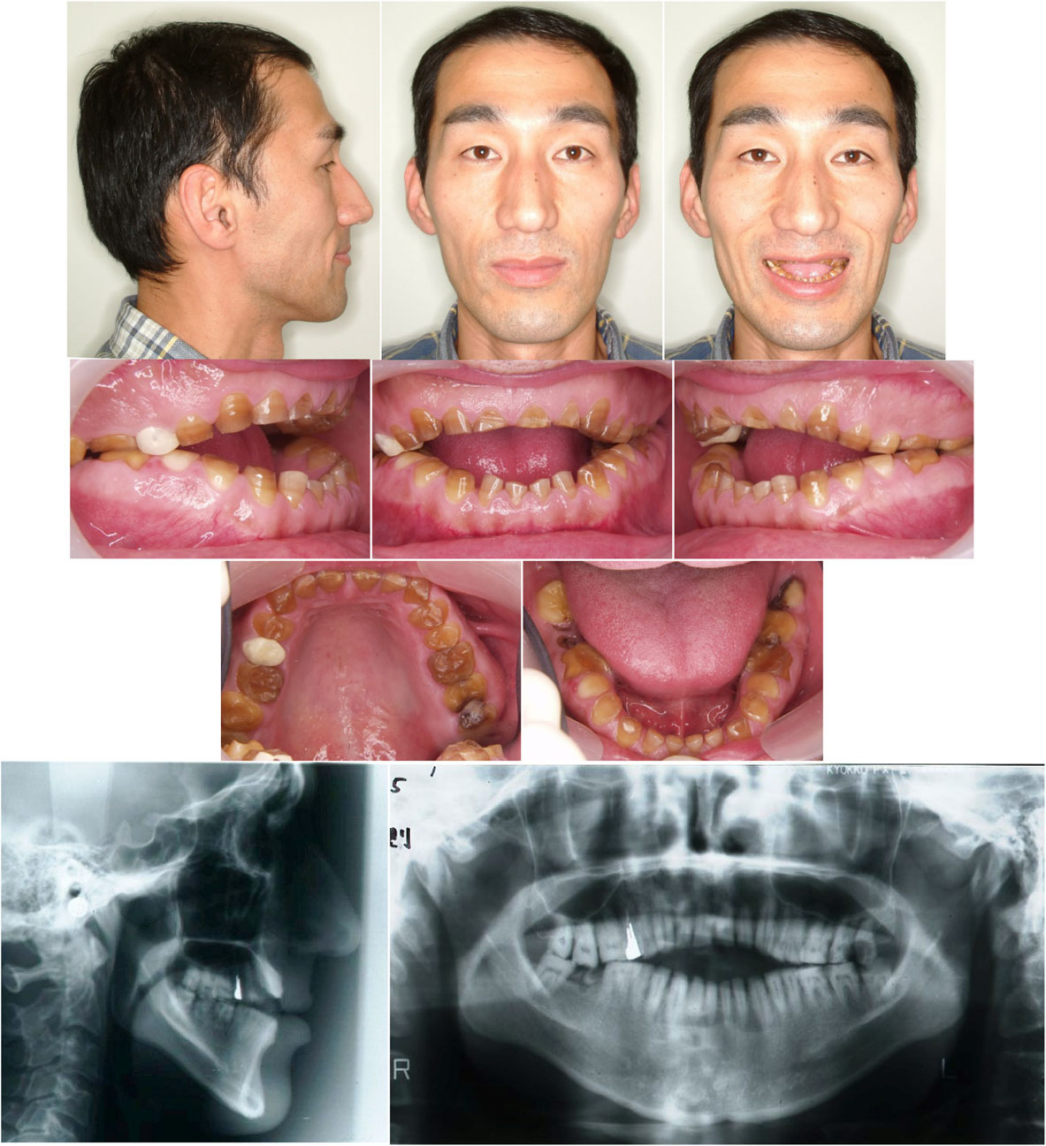
Pretreatment facial and intraoral photographs, lateral cephalogram and panoramic radiograph

Molar relationships were Angle Class I on both sides. All erupted teeth showed severe AI without loss of congenital teeth (Fig. 1). Anterior open bite of − 10.0 mm was observed between the edges of the upper and lower central incisors. The upper dental midline shifted 2.0 mm to the right relative to the facial midline, and the lower dental midline shifted 0.5 mm to the left relative to the facial midline.

Clinical and radiographic examinations revealed a stump lesion on the patient’s lower right second molar and caries lesions on his upper right first premolar, upper and lower left first, second and third molars.

Cephalometric analysis revealed a skeletal Class I jaw-base relationship (Table 1). The mandibular plane and gonial angles were larger than historical values for Japanese control subjects [7], indicating a high mandibular plane angle. The maxillary incisors showed an average degree of inclination, but the mandibular incisors were inclined lingually.

**Table 1:** Cephalometric summary

Variable	Japanese norm^a^	SD	Pretreatment	Posttreatment	Postretention
			37 y 6 mo	38 y 9 mo	46 y 6 mo
Angles (°)					
ANB	3.8	2.4	4.9	3.6	3.7
SNA	81.5	3.3	76.5	76.5	76.5
SNB	78.2	4.0	71.6	72.9	72.8
Mandibular plane-FH plane	28.0	6.1	40.3	37.3	37.4
Gonial angle	120.9	6.5	135.1	135.1	135.1
U1-SN plane	106.0	7.5	105.3	108.4	108.4
U1-FH plane	112.4	7.6	110.0	113.4	113.4
L1-FH plane	56.7	7.8	53.8	57.0	57.0
L1-Mandibular plane	95.2	6.2	85.9	85.2	85.2
Interincisal angle	124.2	8.6	123.8	123.7	123.7
Occlusal plane	15.5	4.2	18.1	19.3	19.3
Lines (mm)					
PTM-ANS/palatal plane	56.4	3.4	59.6	59.6	59.6
PTM-A/palatal plane	51.7	3.8	53.7	51.7	51.7
Ar-Go	53.2	5.7	53.4	53.4	53.4
Ar-Me	115.6	6.8	120.3	120.3	120.3
Go-Me	76.6	4.4	75.4	75.4	75.4
Overjet	3.3	1.0	1.0	2.0	2.0
Overbite	3.3	1.7	−10.0	2.0	2.0

^a^Wada et al. [7])

### Treatment objectives

The patient was diagnosed as a skeletal open bite with severe AI of all teeth erupted, a skeletal Class I jaw-base relationship, and high mandibular plane angle. The treatment objectives were: (1) to correct the anterior open bite and establish ideal overjet and overbite; (2) to achieve acceptable occlusion with good functional Class I occlusion; and (3) to recover the shape of the collapsed teeth with AI by prosthodontic treatment in order to prevent further wear and sensitivity. The treatment was planned as follows:

- 3.5 mm impaction of the posterior maxillary segments (bilateral second premolar, first and second molars) by compression osteogenesis
- Minimal extrusion of the anterior teeth to correct severe open bite
- Establishment of an ideal occlusal relationship through prosthetic restoration.

### Treatment procedure

**Fig. 2:**
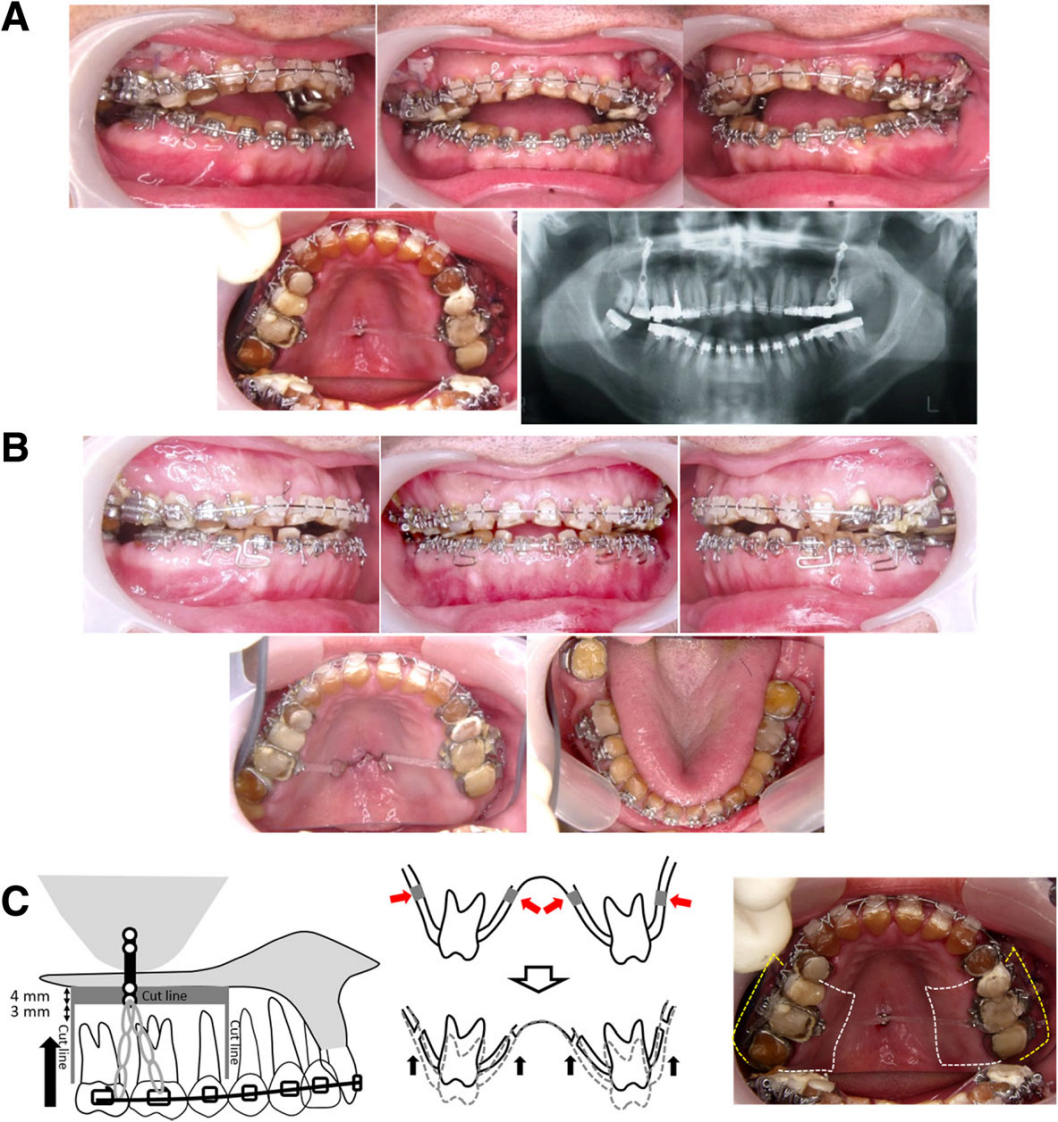
**a** Intraoral photographs and panoramic radiograph taken after two-staged corticotomy at maxillary posterior segments and anchor plate placements. **b** Intraoral photographs taken 8 months after initiation of multibracket treatment. **c** Schematic illustrations of the corticotomy and use of anchor plate. Red arrows indicate corticotomy lines. White dotted lines indicate the first corticotomy, and yellow dotted lines the second corticotomy

After the upper and lower left third molars and the lower second molar were extracted, 0.018″-slot pre-adjusted edgewise appliances were placed on both arches. After leveling, corticotomy was performed under local anesthesia with intravenous sedation. Surgery was performed on an outpatient basis (in two stages to avoid bone necrosis) [6]. In the first stage, corticotomy was initiated at the palatal surface of the first and second upper premolars with a mucoperiosteum incision on the alveolar ridge, 3 mm above the tooth root apices (Fig. 2). A fissure half the width of the desired amount was made with a round bar of 4 mm in diameter through the cortical plate of bone surrounding the teeth. The anchor plate was placed onto the center of the hard palate. The second corticotomy at the buccal site was performed 3 weeks after the first corticotomy (Fig. 2). The mucoperiosteal flap was abraded beforehand to visualize the area of the corticotomy and to ensure that the procedure was carried out in accordance with previously corticotomized regions. The anchor plates were bilaterally fixed to the zygomatic buttress. Then, elastomeric chains were added to move the corticotomized bone/teeth segments 3.5 mm superiorly. After 1 month, posterior maxillary segments were moved superiorly, which ultimately resulted in correction of the skeletal open bite.

After 8 months of postoperative orthodontic treatment, overbite was improved by − 2.0 mm, and molar relationships were maintained as a Class I relationship (Fig. 2). Prosthodontic treatment was initiated to protect dentin and establish stable occlusion. After 5 months of prosthodontic preparation, all edgewise appliances were removed, and provisional crowns were set on all teeth with AI. The total active treatment period was 16 months.

### Treatment outcomes

**Fig. 3:**
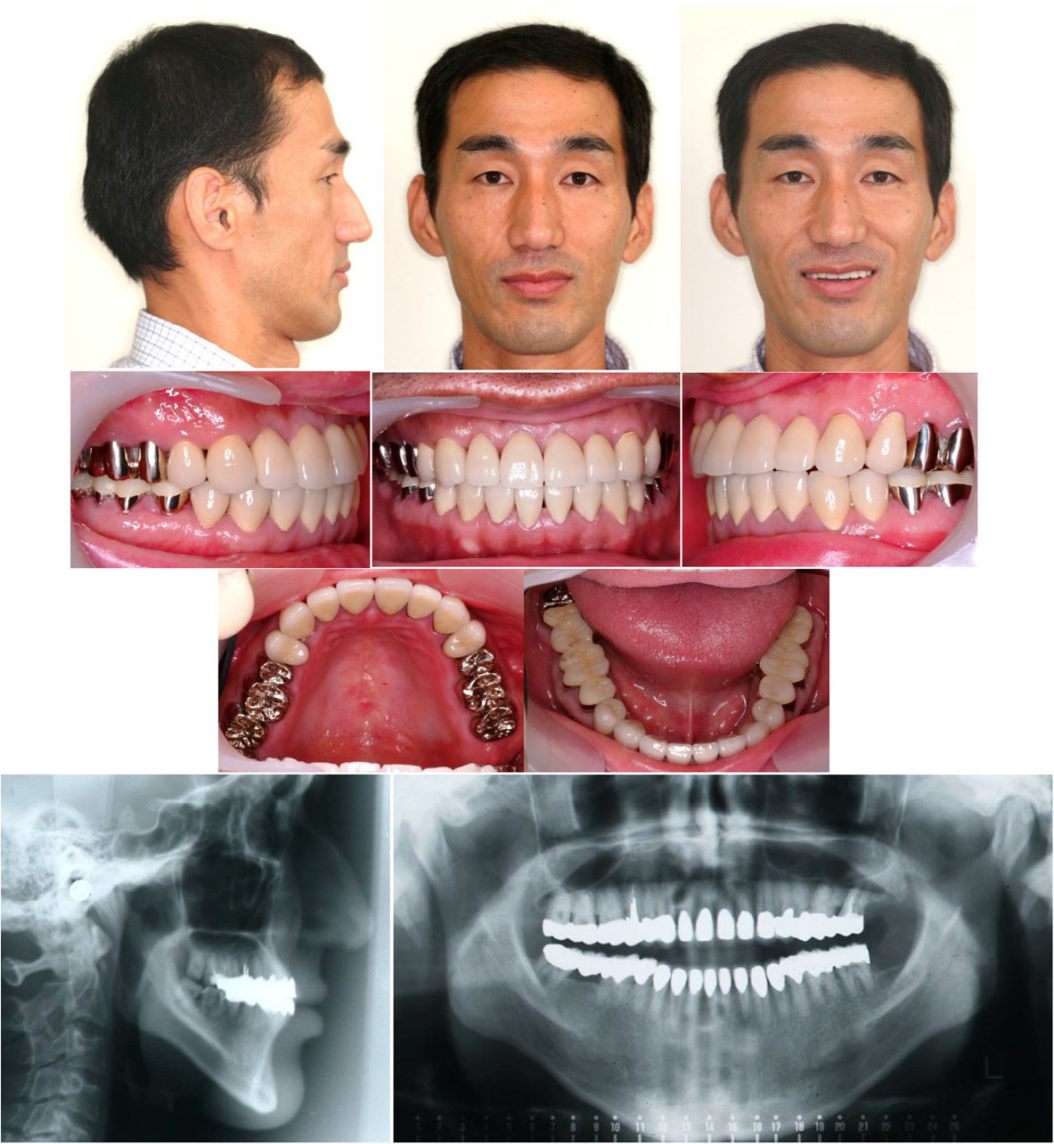
Posttreatment facial and intraoral photographs, lateral cephalogram and panoramic radiograph

On facial photographs obtained after treatment, anterior lower facial height was reduced, resulting in a balanced facial profile (Fig. 3). The anterior open bite was corrected, and the occlusion was much more stable and acceptable, with Class I canine and molar relationships. The overjet and overbite were + 2.0 mm and + 1.5 mm, respectively. When the patient smiled, approximately one-third of the maxillary central incisors were properly exposed. Panoramic radiograph showed that root parallelism was achieved (Fig. 3).

**Fig. 4:**
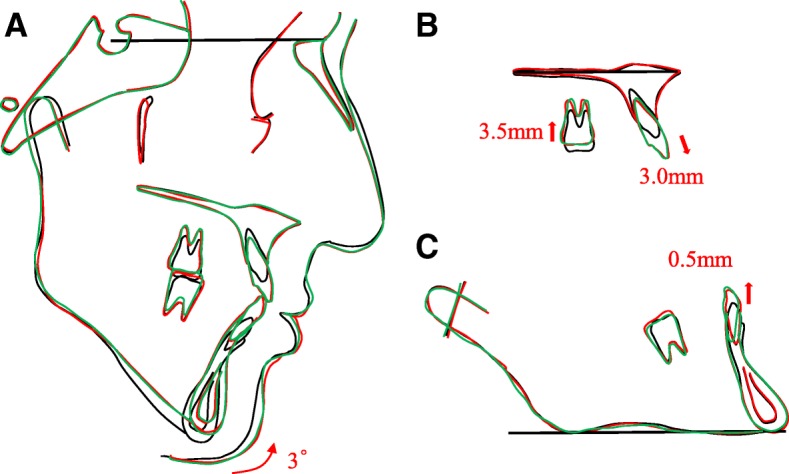
Cephalometric tracings at pretreatment (black line), posttreatment (red line), and eight-year postretention (green line), superimposed on **a**, Sella-Nasion plane at Sella; **b**, the anterior palatal contour; and **c**, the mandibular plane at menton

Post-treatment cephalometric evaluation showed a skeletal Class I jaw base relationship (ANB 3.6°) (Table 1, Fig. 4). The mandible was rotated 3.0° counter-clockwisely (MP-FH plane 37.3°). The upper first molars were intruded 3.5 mm toward the palatal plane. The maxillary and mandibular central incisors were extruded 3.0 mm and 0.5 mm, respectively. These factors contributed to the maintenance of an acceptable interincisal relationship.

**Fig. 5:**
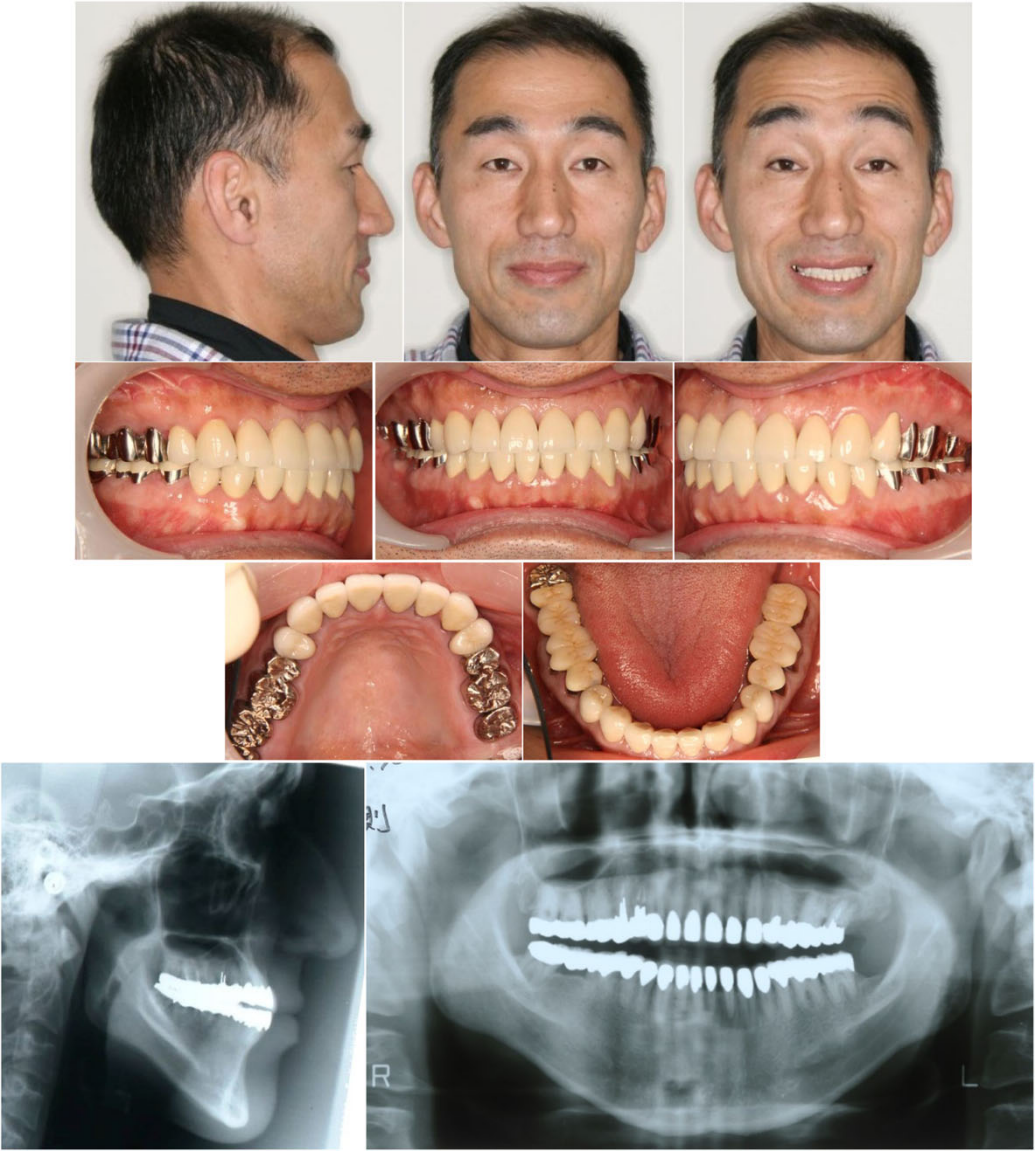
Facial and intraoral photographs, lateral cephalogram and panoramic radiograph at eight-year postretention

After 8-year retention, the occlusion was stable, and a good facial profile was maintained (Fig. 5). Panoramic radiograph revealed no or less changes in alveolar bone level and root parallelism (Fig. 5). Cephalometric analysis showed only minor changes in maxillary and mandibular position, which did not result in relapse (Fig. 4).

## Discussion

AI, a hereditary genetic defect that affects tooth enamel, is heterogeneous in clinical phenotype as well as in genetic etiology. Recently, Prasad et al. [8] confirmed the phenotype-genotype correlation between various forms of AI and underlying genetic mutations. To date, mutations in 15 genes including *AMELX*, *ENAM*, and *AMBN*, have been shown to cause nonsyndromic AI [8]. Phenotypically, this disease is broadly classified into hypoplastic vs. hypomineralized AI. Hypoplastic AI is characterized by extremely thin or no enamel, pitted enamel, or enamel with grooves. Hypomineralized AI is characterized by soft enamel [8].

Our case showed very severe and extensive loss of tooth enamel, tooth sensitivity and excessive attrition, leading to short clinical crowns. Then, all erupted teeth showed a yellowish color due to severe enamel hypoplasia. Dental radiographs showed reduced enamel throughout the permanent dentition. This implies this case was classified as hypoplastic AI, while this case was not associated with any particular genetic mutation. In mild or moderate cases of AI, a simple and effective treatment approach involves allowing the natural stratification of composite resins to mask deficient enamel formation and mimic the substrate’s natural appearance [9]. However, this case showed crown collapse throughout all erupted permanent teeth with vertical discrepancy. We therefore decided to combine surgical orthodontic and prosthetic treatments.

Prosthetic restoration without orthodontic treatment might shorten the total treatment period; however, the prosthodontic treatment of molars is subject to clockwise rotation of the mandible, leading to the impairment of anterior open bite and long face. Complex two-jaw surgery is considered to be a relatively routine intervention for patients with severe anterior open bite, and the combination of simple orthognathic surgery with a skeletal anchorage system for tooth movement boasts several advantages: reduction of surgical invasion and autorotation of mandible. However, in both treatments, surgery under general anesthesia is an invasive procedure that may lead to considerable discomfort for patients, sometimes necessitating intense postoperative care.

Molar intrusion with skeletal anchorage has been established as a reliable treatment alternative. For example, compression osteogenesis which is an osteoplasty technique based on the phenomenon of distraction osteogenesis, was developed as an outpatient treatment for anterior open bite. This method of molar intrusion is less invasive, involves less psychological stress, and results in less postoperative discomfort, compared with two- or one-jaw osteotomy surgery as an inpatient. Yokoo et al. [10] demonstrated 2-stage operation with 3-week interval was beneficial because of the risk of necrosis of the bone fragments. In addition, Choo et al. [11] indicated that a 2-stage perisegmental corticotomy can be less technique sensitive with low risks of tissue damage and more predictable outcome. After considering all available evidence, we chose to proceed with compression osteogenesis with two-stage corticotomy in the posterior maxilla.

Compared with surgical osteotomy, corticotomy is less invasive and involves less discomfort. The advantage of corticotomy-facilitated orthodontic treatment is accelerated tooth movement, which decreases treatment time. With the corticotomy procedure, bone can be augmented, thereby preventing periodontal defects, which may appear in cases with thin alveolar bone. No postoperative complications were reported [6, 12].

Compression osteogenesis also allows for postoperative adjustment of bone/tooth segments to ideal position using a gradual compressive force over a shortened treatment period. In this case, it took only 2 months to intrude the upper molars by 3.5 mm (without root resorption). Total active treatment time was 16 months, which is significantly shorter than the treatment period necessary for conventional orthodontic treatment. No relapse was observed at 8-year follow-up.

## Conclusions

Our results demonstrate long-term stability after combined treatment with orthodontics, compression osteogenesis, and prosthodontics in a patient with severe anterior open bite and AI. Compression osteogenesis may facilitate treatment of jaw deformities with vertical discrepancy and other complex problems.

## Abbreviation

AI: Amelogenesis imperfecta
